# Health-related quality of life in cancer survivors 5 years after total thyroidectomy: a population-based study

**DOI:** 10.1210/jendso/bvag228

**Published:** 2026-10-01

**Authors:** Hayat Mcheik, Julien Mancini, David Taïeb, Sandrine Dabakuyo, Agnès Dumas, Anne-Déborah Bouhnik

**Affiliations:** Aix-Marseille Université, INSERM, IRD, ISSPAM, SESSTIM, Sciences Economiques & Sociales de la Santé & Traitement de l'Information Médicale, 13005 Marseille, France; Aix-Marseille Université, INSERM, IRD, ISSPAM, SESSTIM, Sciences Economiques & Sociales de la Santé & Traitement de l'Information Médicale, 13005 Marseille, France; Aix-Marseille Université, APHM, Inserm, IRD, SESSTIM, Sciences Economiques & Sociales de la Santé & Traitement de l’Information Médicale, ISSPAM, Hop Timone, BioSTIC, Biostatistique et Technologies de l’Information et de la Communication, 13005 Marseille, France; Aix Marseille Université, APHM, Inserm, IRD, SESSTIM, Sciences Economiques & Sociales de la Santé & Traitement de l’Information Médicale, ISSPAM, Hop Timone, Médecine Nucléaire, 13005 Marseille, France; Georges-François Leclerc Centre, Breast and Gynaecologic Cancer Registry of Cote d’Or, INSERM U1231, UNICANCER, Dijon, 21079 Bourgogne-Franche-Comté, France; Aix-Marseille Université, INSERM, IRD, ISSPAM, SESSTIM, Sciences Economiques & Sociales de la Santé & Traitement de l'Information Médicale, 13005 Marseille, France; Aix-Marseille Université, INSERM, IRD, ISSPAM, SESSTIM, Sciences Economiques & Sociales de la Santé & Traitement de l'Information Médicale, 13005 Marseille, France

**Keywords:** thyroid cancer, health-related quality of life, population-based study, survivorship

## Abstract

**Context:**

Long-term data on thyroid cancer survivors (TCS), a population experiencing persistent physical and mental health-related quality of life (HRQoL) impairment, are limited.

**Patients and Methods:**

Thyroid cancer survivors diagnosed in 2010-2011 were randomly selected from the French National Insurance database. Clinical data on consenting participants were retrieved from medical files while Physical and Mental Component Summary (PCS/MCS) HRQoL scores were calculated using the SF-12 questionnaire 2 and 5 years post-diagnosis. Health-related quality of life impairment was defined using reference values for the French general population. We compared HRQoL with both the general population and patients with other common cancers having good prognoses. Logistic regression identified factors associated with impaired HRQoL.

**Results:**

Of the 130 survivors reporting HRQoL 5 years post-diagnosis, 83.8% were women, 97.6% had undergone total thyroidectomy, and mean age was 40 years. Physical and mental HRQoL impairment was observed in 49% and 41% of participants, respectively, figures comparable to or exceeding those in survivors of other common cancers with good prognoses. Age- and sex-standardized PCS and MCS z-scores were −0.34 [−0.52 to −0.16] and −0.51 [−0.69 to −0.34], respectively (*P* < .001). The PCS and MCS scores remained stable (71.8% and 75.3% of the sample, respectively), with 11.8% and 5.9%, respectively, experiencing a deterioration. Lower educational levels, anxiety, and activity limitation were associated with physical HRQoL impairment, whereas male sex was associated with mental impairment. Body image discomfort was associated with impairment for both domains.

**Conclusion:**

Persistent HRQoL impairment among TCS 5 years post-diagnosis highlights the need for personalized, long-term care strategies.

The global number of thyroid cancer survivors (TCS) had risen substantially in recent decades, in particular among adolescents and young adults (AYAs) (1). This increase has largely been driven by advances in diagnostic technologies that enable more frequent and earlier detection of small, low-risk tumors (2). According to 2022 GLOBOCAN data, differentiated thyroid cancer (DTC) is the second most diagnosed cancer among female AYAs, after breast cancer, and the most common cancer among male AYAs (2).

Europe accounts for a substantial share of the global thyroid cancer (TC) burden. In 2022, there were 78 552 new cases (9.6% of global incidence) and 5902 deaths (12.4% of global mortality), with an estimated 5-year prevalence of 305 021 cases (10.5% of global prevalence). In France, the focus country of the present study, there were 9618 new TC cases and 476 related deaths in 2022, with an estimated 5-year prevalence of 37 887 cases (57.8 per 100 000 population) (3).

Differentiated thyroid cancer accounts for 90% of cases worldwide (4); it is treated surgically (partial or total thyroidectomy), with or without radioactive iodine (RAI), followed by thyroid hormone replacement therapy (5). The 10-year global DTC survival rate is 92% (6). Despite this favorable situation, a growing body of evidence suggests that TCS, especially AYAs and other persons under 45 years of age, often experience a lower health-related quality of life (HRQoL) compared with survivors of cancers with lower survival rates (7), marked by persistent fatigue, psychological distress, fear of recurrence, and metabolic disturbances (8). Fatigue is the most commonly reported physical symptom, with no significant difference between persons surviving <5 years and those surviving >15 years (9). These 4 factors have been linked to treatment effects, negative perceptions of illness, and concerns about one's body image following surgery (10).

Although physical exercise-based interventions and psychosocial support programs have shown some potential in alleviating fatigue and psychological distress (11), no validated interventions currently exist to overcome the physical and mental challenges that TCS face (12). Despite advances in TC care, especially for DTC, there is considerable heterogeneity among patients in terms of the extent of surgery (ie, less vs more invasive), RAI use, target thyroid-stimulating hormone (TSH) levels while receiving hormone replacement, and surveillance protocols (13, 14). Concerning the latter, recent guidelines from both the American Thyroid Association (ATA) and the European Society for Medical Oncology (ESMO) emphasize a personalized strategy, adjusting follow-up based on each patient's risk of recurrence and treatment response (15, 16). Recent DTC management guidelines underline the importance of considering treatment-related side effects and patient-reported outcomes and highlight the need for additional high-quality research (15). In this context, research on long-term outcomes could provide valuable insights to guide future clinical practice and policies (17). The French national population-based VICAN (“*VIe après CANcer*”) surveys were specifically designed to collect such data (18).

The present study used data from these surveys to (1) compare the HRQoL of TCS to that of individuals with other common cancers with good prognoses, and with the general population, (2) assess temporal changes in HRQoL in this population over time, and (3) identify sociodemographic, clinical, and psychosocial factors associated with impaired HRQoL 5 years after diagnosis.

## Methods

### Study design

Coordinated by the French National Cancer Institute (INCa), the 2 national population-based surveys VICAN 2 and VICAN 5 were implemented in 2010-2011 and 2015-2016, respectively. They aimed to assess the living conditions and HRQoL of cancer survivors, including TCS, 2 (VICAN 2) and 5 (VICAN 5) years after diagnosis. Both surveys included a longitudinal sample of survivors who were assessed at both time points. However, for the VICAN 5 survey, an additional independent sample of survivors was also assessed. The inclusion of this complementary sample was an integral component of the survey design, the rationale being to ensure a larger study population for analysis at the 5-year time point (19). The present study used data from both surveys. The methods and results are reported in accordance with the STROBE checklist (20).

### STUDY population

The study populations for the 2 VICAN surveys were randomly selected from the French National Health Insurance Claims Database (*SNIIRAM*). The methodologies used and data collection procedures implemented are described in detail elsewhere (19). Briefly, eligible individuals met the following criteria: resident in France, aged 18 to 82 years at the time of cancer diagnosis, covered by the national health insurance system, and diagnosed with cancer in 2010 or 2011. The surveys focused on 12 specific cancer sites, including TC. Informed consent was mandatory for inclusion.

A 2-step weighting procedure was used in both surveys to ensure that study samples reflected the target population. First, sampling weights were calculated based on age, the type of health insurance scheme (general, agricultural, self-employed), and cancer site, using the inverse of the sampling fraction for each stratum. Second, to adjust for non-response, survey participants’ and decliners’ available characteristics (age, sex, cancer site, socioeconomic deprivation index of the place of residence, access to a general practitioner, and urbanicity of the place of residence) were compared. These differences were then incorporated into a calibration procedure using raking ratio adjustments (19).

### Data collection

Survey data collection involved 3 components: (1) data extraction from the *SNIIRAM* database, which included information on healthcare utilization and reimbursed medications; (2) extraction of clinical data on cancer characteristics (eg, tumor size) and treatments from medical files, the latter being provided by participants’ treating physicians; and (3) individual patient surveys conducted via Computer-Assisted Telephone Interviewing (CATI), which collected data on sociodemographics, health status, HRQoL (using psychometric tools), cancer-related symptoms (eg, fatigue and pain) and lifestyle habits (18).

### Outcome

For the present analysis, the outcome was survivor HRQoL, assessed using the SF-12v2, which provides 2 summary measures: the Physical Component Score (PCS) and the Mental Component Score (MCS) (21). Specifically, raw SF-12v2 item responses were first scored according to the standard SF-12v2 scoring algorithm described in the SF-12v2 User Manual (Ware J.) (22), using the US norm-based scoring procedure (mean = 50, SD = 10). Each score ranges from 0 to 100, with higher values indicating better HRQoL. To enable standardized comparisons with the French general population, PCS and MCS z-scores were then calculated using age- and sex-specific reference values from the French general population (23).

As HRQoL varies according to age, sex, and country (24), these age- and sex-stratified French reference values were used for 2 purposes: (1) to enable comparisons with other common cancer sites with good prognoses and (2) to define HRQoL impairment at the individual level. Impaired PCS and MCS were defined as scores were below the 25th percentile of the corresponding age- and sex-specific distribution in the French general population; scores equal to or above this threshold were considered normal.

### Explanatory factors

#### Sociodemographic characteristics

Sociodemographic characteristics from the CATI questionnaire included family status, dichotomized into “No dependent children” vs “At least one dependent child”; education level, dichotomized into “Less than high school diploma” vs “High school diploma or higher”; and employment status at the time of the survey, dichotomized into “Active” vs “Inactive” (ie, unemployed, student, retired, disabled, homemaker, or on leave from work). We also measured patients’ self-perceived financial situation using the question “Would you say that financially…?” with the following answer options: “You are comfortable, “You get by”, “You just make ends meet; you need to be careful”, “It's difficult to get by”, and “You can’t get by without going into debt”. We dichotomized these responses into “stable” (combining the first 2 options) vs “difficult” (combining the last 3 options).

#### Clinical data

Clinical data from the *SNIIRAM* medico-administrative database and medical files included information on TC diagnosis, specifically the pathological report (ie, differentiated cancer: papillary or follicular; medullary cancer), absence (M0)/presence (M1) of distant metastases, and lymph node involvement (N+), with (N0) indicating no involvement. We used the American Joint Committee on Cancer TNM tumor staging system to describe tumor size and local invasion, categorized as T1–T4; a higher T category indicates a larger tumor and/or greater local extension. Disease stage was recorded as I, II, or III/IVa/IVb, with higher stages corresponding to more advanced disease.

Surgical procedures (ie, total or partial thyroidectomy), lymph node dissection and subsequent treatments (ie, radiotherapy, RAI, chemotherapy, thyroid hormone replacement therapy), were recorded as “yes” or “no” by combining data from all 3 data sources.

Data from medical files provided by physicians were the primary data source and were available for 92.4% of participants. Missing medical file data were handled using *SNIIRAM* data (2.1%) and data from the CATI questionnaires (5.5%). With regard to surgical procedures, data were missing for only 2 cases after combining all 3 sources.

#### Body weight and body mass index

Pre-diagnosis and post-diagnosis body weights (BW) were self-reported. Three indicators were used to assess variations in BW over time: weight loss (ie, losing ≥ 5 kg), stable weight (weight fluctuation ± 5 kg), and weight gain (ie, gaining ≥ 5 kg) (25). We also used the body mass index (BMI), which equals BW/height (meters)^2^, to classify body type according to 4 categories: underweight (BMI < 18.5 kg/m^2^), normal weight (18.5 kg/m^2^ < BMI < 24.9 kg/m^2^), overweight (25 kg/m^2^ < BMI < 29.9 kg/m^2^), and obesity (BMI > 30 kg/m^2^).

#### Cancer-related fatigue and activity limitation

To assess clinically relevant fatigue, we used the 3-item fatigue subscale from the EORTC QLQ-C30 questionnaire, which generates scores ranging from 0 to 100. Fatigue was coded as a binary variable: “Yes” for scores ≥ 40, indicating clinically relevant fatigue, and “No” for scores < 40, indicating no clinically relevant fatigue (26).

Participants were also asked whether they had experienced fatigue attacks, felt weak, tired, or needed to rest during the previous week. Each of these 4 questions used a 4-point Likert scale ranging from “Very often” to “Never”. For the present analysis, we dichotomized these responses into 2 categories: Yes (combining “Very often” and “Often”) and No (combining “Rarely” and “Never”).

Activity limitation was evaluated using the Global Activity Limitation Indicator (GALI), which is a well-established instrument measuring participation restriction (27). Participants answered the following question: “Have you been limited in activities that people usually perform for at least the past six months due to a health problem? (yes, severely limited; yes, limited but not severely; no, not limited at all)” (18).

#### Cancer treatment-specific sequelae and body image perception

We also documented episodes of self-reported, cancer treatment-specific side effects in the 7 days prior to the survey, including hot flashes, muscle cramps, neck or shoulder mobility issues, and swelling in one arm, with 4 answer options (none, a few, quite a few, and a lot). Of note, these sequelae reflect a person's global health status and the potential consequences of disease and treatment; they are not specific to TC treatment complications. For the present analysis, we dichotomized responses into 2 categories: Yes (combining “a lot” and “quite a few”) and No (combining “a few” and “none”).

Additionally, 4 questions taken from the Hopwood questionnaire assessed how treatment impacted self-image in terms of physical appearance, sexual attractiveness, and changes related to both illness and to treatment (28). Specifically, participants were asked whether, in the previous 7 days, they had (1) “felt embarrassed by their appearance”, (2) “avoided others due to negative feelings about their appearance”, (3) “felt that treatment had made their body less whole”, and (4) “been dissatisfied with the appearance of post-treatment scars”. Each question had a 4-point Likert scale response. For the present analysis, we dichotomized answers into 2 categories: Agree (combining “Strongly agree” and “Somewhat agree”) and Disagree (combining “Somewhat disagree” and “Strongly disagree”). Participants were also asked whether their illness had changed, increased, or decreased their sexual desire.

#### Anxiety and depression

Anxiety and depression were assessed using the Hospital Anxiety and Depression Scale (HADS) (29), which creates a subscale for each condition. These subscales were coded into 3 categories: non-case (0-7), doubtful case (8-10), and definite case (11-21).

#### Analyses

Descriptive analyses were conducted using χ² tests for categorical variables. Standardized z-scores were computed based on the age- and sex-specific distribution in the French general population for each summary score (ie, PCS and MCS) to allow age and sex HRQoL comparisons. Longitudinal changes in HRQoL were evaluated among participants who completed the SF-12v2 at both 2 (VICAN 2) and 5 (VICAN 5) years after diagnosis (ie, not the complementary VICAN 5 sample). At each time point, PCS and MCS were categorized as impaired or normal using the 25th percentile threshold described above. Individual changes over time were then classified as improved (ie, moving from impaired to normal), stable (no change), or deteriorated (moving from normal to impaired) at 5 years post-diagnosis.

Binary logistic regression models were then used to identify factors associated with impaired mental and physical HRQoL. A controlled stepwise selection procedure, combining backward and forward approaches, was applied, progressively removing variables that were not statistically significant (*P* > .05). We used IBM SPSS Statistics version 27 (IBM Corp., Armonk, NY, USA) for the statistical analyses.

## Results

The TCS selection process for our current analysis is presented in Fig. 1. Overall, 917 TCS (439 from VICAN 2 and 478 from the additional sample) were eligible for participation in VICAN 5. After weighting and selection procedures, as well as exclusion of those who did not (fully) complete the CATI questionnaire, a total of 146 persons (91 from the VICAN 2 sample and 55 from the additional sample) comprised our study sample, corresponding to an overall inclusion rate of approximately 15.9%. Participants diagnosed with cancer other than DTC were subsequently excluded, resulting in a final study sample of 130 survivors. No significant differences were observed between eligible patients who responded to VICAN 2 and 5 and those who did not, with respect to gender, age, tumor stage, or socioeconomic deprivation index of the place of residence.

**Figure 1:**
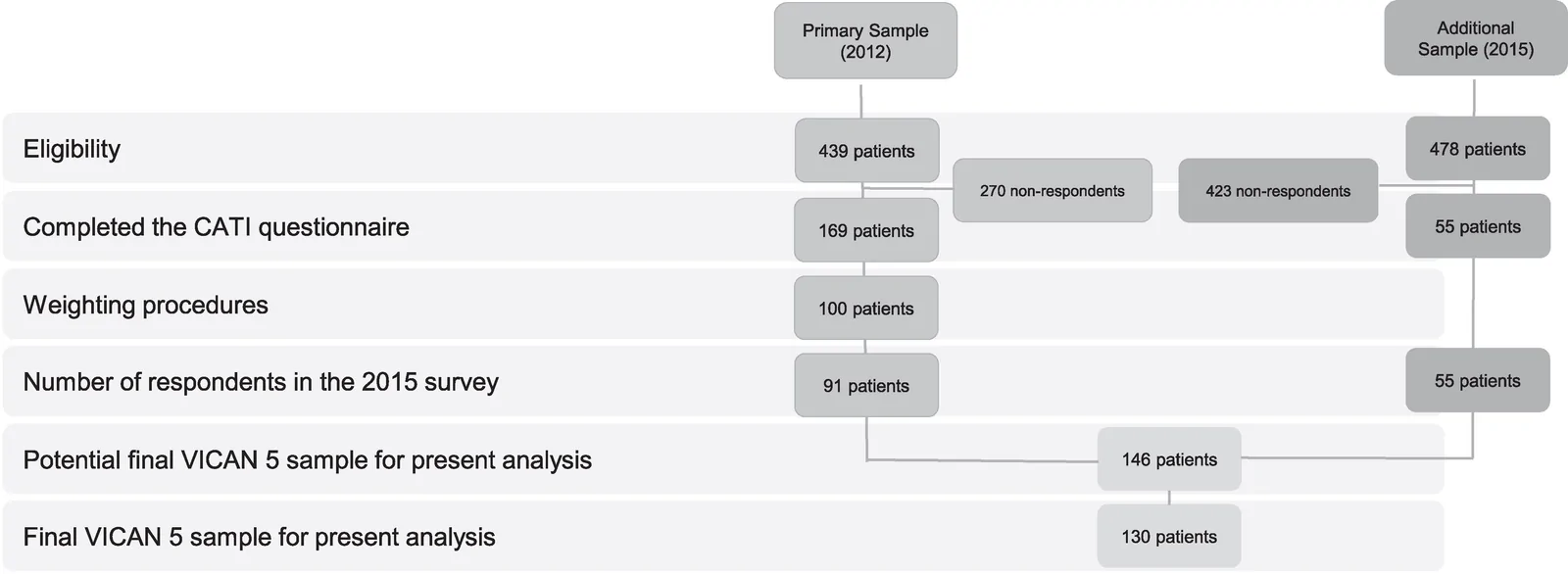
Flowchart of thyroid cancer survivor selection for participation in VICAN 5 and for the present analysis (30).

The weighting procedures used a stratified random sampling design with non-proportional allocation. Sampling weights were first computed as the reciprocals of the selection probabilities within each stratum (30).

### Study sample

Four-fifths (83.8%) of our study sample were female. Mean age at diagnosis was 40 years. At the time of the VICAN 5 survey, 40.0% had at least one dependent child, 64.6% had completed at least high school, 82.3% were employed, and 60.0% reported having a difficult financial situation. With regard to clinical characteristics, 96.9% of the patients had DTC, 64.6% had no distant metastases (M0), and 2.3% had distant metastases (M1), while 33.1% had unknown metastatic status; regional nodal involvement was absent in 36.9% (N0) and present in 32.3% (N+), with 30.8% unknown. Slightly over half (53.8%) had a T1 TNM stage, 20.8% T2, and 22.3% T3 or T4. Three-quarters (74.6%) of the sample were diagnosed with stage I cancer disease. With respect to clinical interventions, 97.6% had undergone total thyroidectomy, 55.9% had undergone lymph node dissection, 2.4% had received radiotherapy, 86.0% had undergone RAI therapy, 3.8% had received chemotherapy, and 96.2% were currently on thyroid hormone replacement therapy. Five years after diagnosis, 49.6% and 41.2% of the participants had impaired PCS and MCS, respectively. Furthermore, 40.3%, 59.2%, and 46.6% reported weight gain, clinically significant fatigue, and activity limitation, respectively. With regard to body image, 33.1% felt uncomfortable about their appearance, 13.8% avoided others because of negative feelings related to their body appearance, 33.8% felt their body was less whole after treatment, and 13.1% were dissatisfied with the appearance of post-treatment scars. Two-fifths (32.6%) reported a decrease in sexual desire since diagnosis, 63.6% anxiety symptoms, and 16.3% depressive symptoms (Table S1 (31)).

### Study outcome: HRQOL

#### Comparison of HRQoL between TCS and survivors of other common cancers with good prognoses

As shown in Fig. 2, participants presented substantial PCS and MCS impairment, which was comparable to or exceeded than that observed in survivors of other common cancers with good prognoses. Specifically, approximately half (49.6%) reported impaired PCS, a proportion similar to breast cancer survivors, but higher than that observed among prostate, colorectal, and melanoma survivors. MCS impairment affected over 40% of the sample, exceeding levels reported for the same common cancer sites with good prognoses.

**Figure 2:**
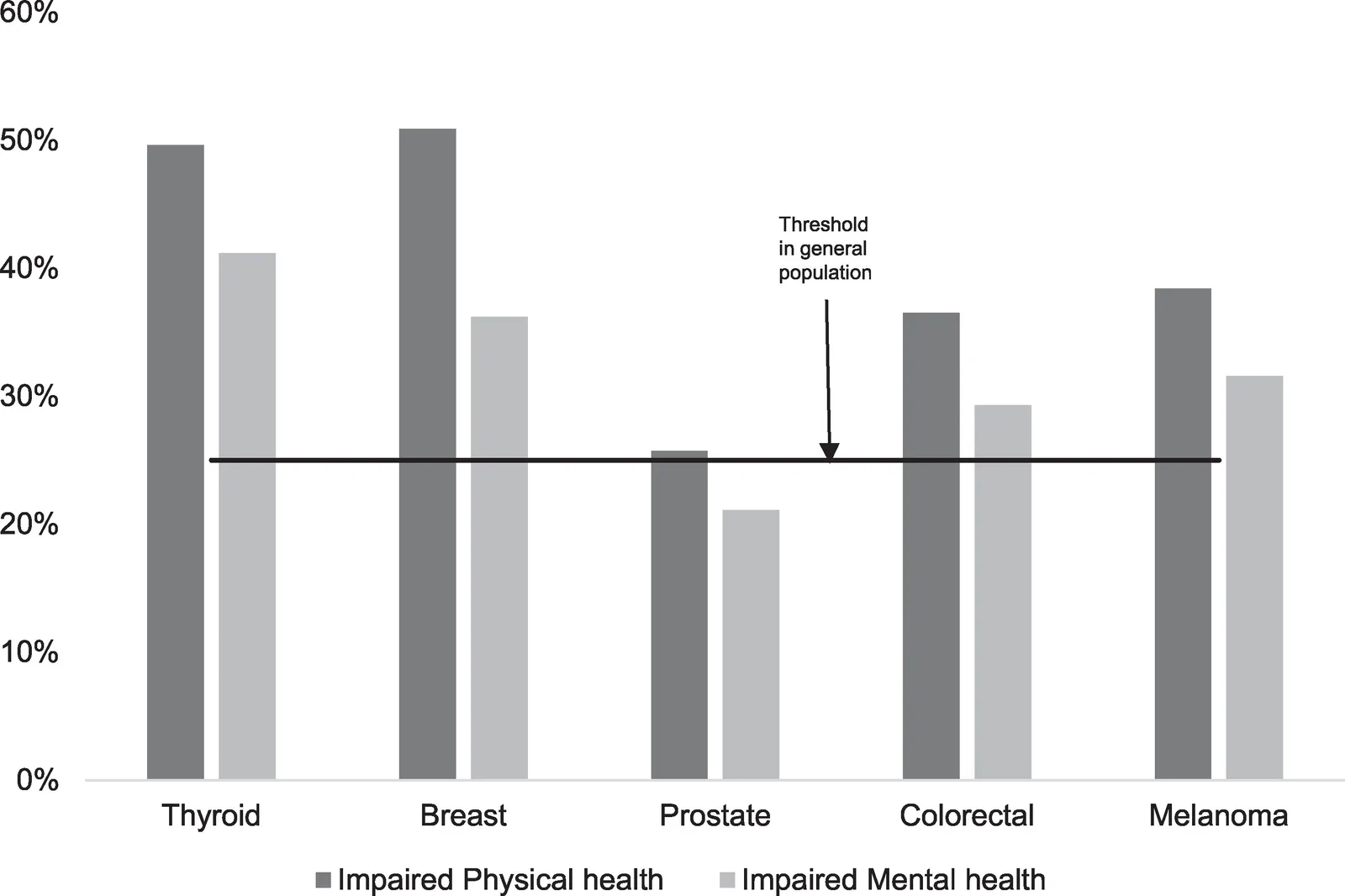
Comparison of physical and mental health impairment in thyroid cancer survivors and survivors of other common cancers with good prognoses (N = 130 with thyroid cancer, 1192 with breast cancer, 447 with prostate cancer, 517 with colorectal cancer, and 285 with melanoma).

#### Comparison of HRQoL scores between TCS and the general population

In the overall sample, the standardized mean physical and mental z-scores [95% CI] were −0.34 [−0.52 to −0.16] and −0.51 [−0.69 to −0.34], respectively; these were significantly lower than those of the general French population (*P* < .001 for both). Compared with the latter, PCS impairment was modest, while MCS impairment was moderate and clinically significant.

#### PCS and MCS evolution among TCS at 2 and 5 years post-diagnosis (N = 85)

Figure 3 depicts the evolution of PCS and MCS post-diagnosis among TCS who responded to both VICAN surveys. For the former, 16.5% of this subsample showed improvement (ie, from impaired to normal health), 71.8% remained stable, and 11.8% experienced a deterioration (ie, from normal to impaired health). For the latter, these figures were 18.8%, 75.3%, and 5.9%, respectively.

**Figure 3:**
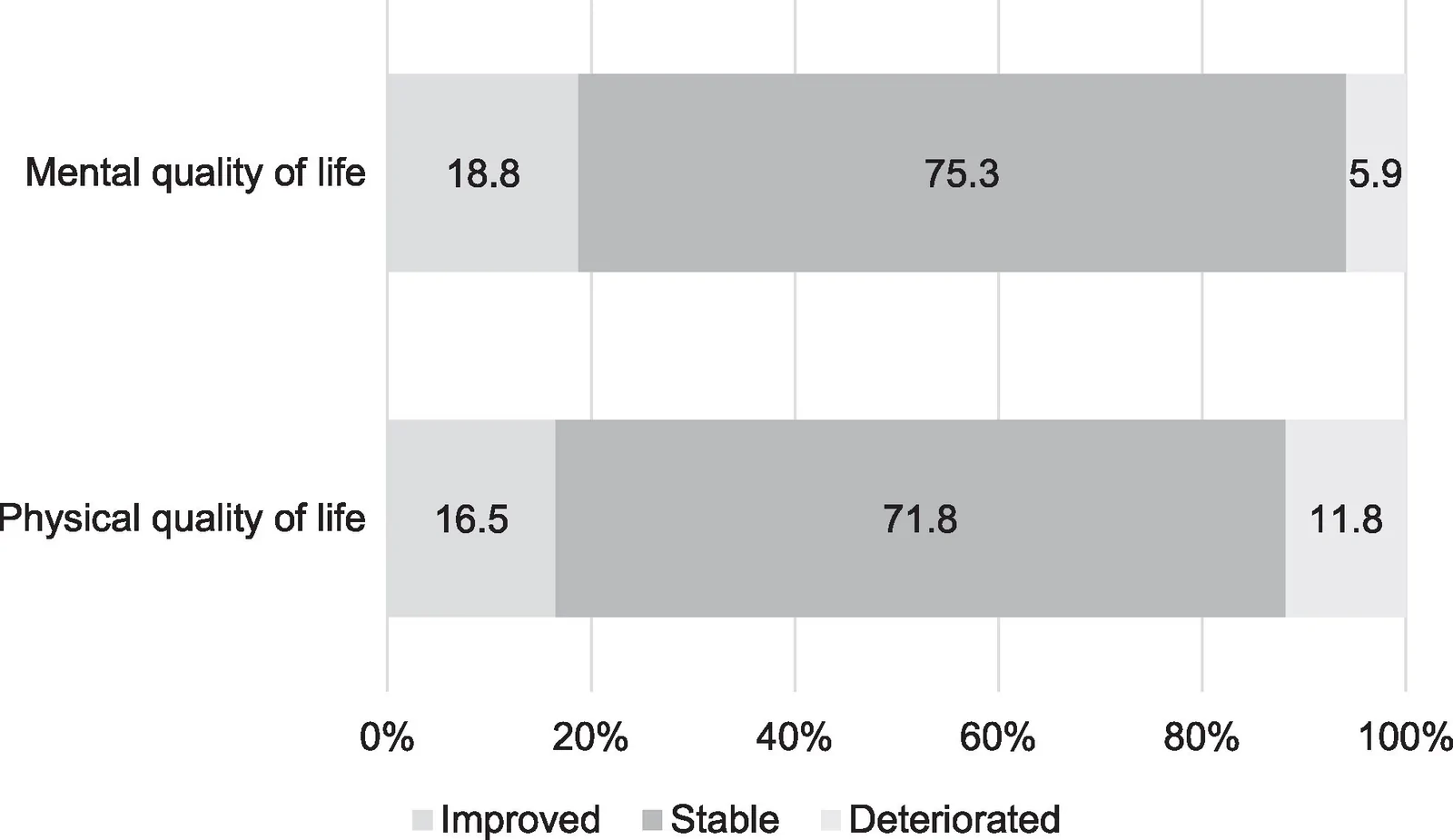
Evolution of the physical and mental domains of health-related quality of life among thyroid cancer survivors 2 and 5 years after diagnosis (N = 85).

#### Factors associated with impaired HRQOL 5 years after diagnosis—univariable analyses

Several factors were significantly associated with low PCS and/or low MCS. For example, the former was linked to having a level of education lower than high school diploma and activity limitation while the latter was associated with having overweight at the time of the survey. In addition, both PCS and MCS were adversely affected by financial difficulties, high fatigue levels and treatment-related symptoms such as hot flashes, muscle cramps, and neck or shoulder movement difficulties. Moreover, anxiety, discomfort with one's appearance, social avoidance due to concerns about one's appearance, feeling less complete, and decreased sexual desire due to illness or treatment were all consistently associated with poorer outcomes for both domains (Table S2 (31)).

#### Factors associated with IMPAIRED HRQOL—multivariable analyses (Fig. 4)

TCS 50 to 59 years old had a significantly higher PCS score compared to those aged between 40 and 49 (95% CI: 0.03-0.78; *P* = .023). Participants with a lower education level were significantly more likely to have a low PCS score (95% CI: 1.73-14.71; *P* = .003). Similarly, those who had activity limitation (95% CI: 1.74-11.86; *P* = .002), individuals who felt discomfort related to their body image (95% CI: 1.41-10.05; *P* = .008), and patients with suspected or confirmed anxiety (95% CI: 1.45-10.36; *P* = .007) were all more likely to have a low PCS score. In contrast, male sex was associated with a lower MCS score than female sex (95% CI: 1.01-10.34; *P* = .045). The odds of having a low MCS score were substantially higher among patients who perceived their body as incomplete after treatment (95% CI: 2.02-11.66; *P* < .001). Participants who reported decreased sexual desire due to their illness had 2.47 times higher odds of having a low MCS score; this association approached statistical significance (95% CI: 0.95-6.10; *P* = .055), suggesting a possible link that warrants further investigation.

**Figure 4:**
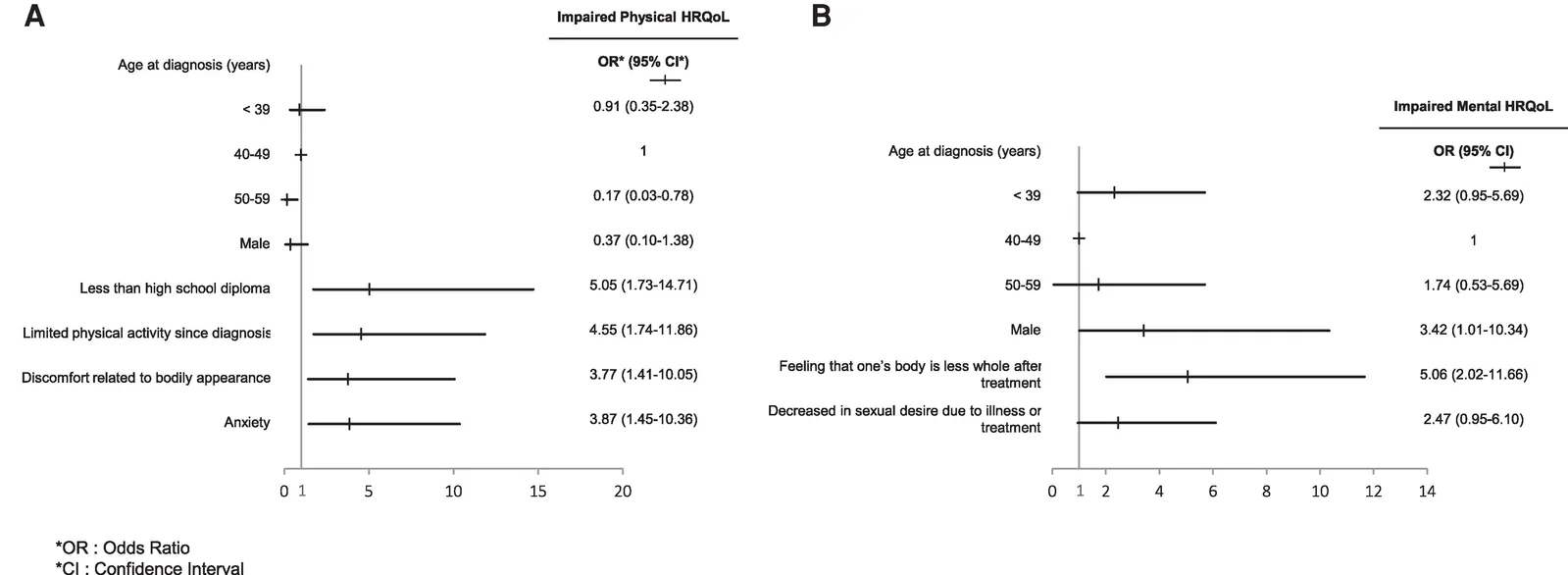
Factors associated with impaired health-related quality of life (HRQoL): (A) impaired physical HRQoL and (B) impaired mental HRQoL 5 years after thyroid cancer diagnosis in 2010-2011, France (n = 130, binary logistic regression).

## Discussion

Our findings indicate that 5 years after diagnosis, a substantial proportion of TCS undergoing total thyroidectomy in the VICAN 5 survey (accounting for 97.6% of our study sample) continued to experience impaired HRQoL, with 49.6% and 41.2% presenting low (ie, <25th percentile threshold) PCS and MCS scores, respectively. Moreover, despite the generally favorable prognosis of TC, nearly half of the sample reported impaired physical HRQoL, a percentage comparable to that observed among breast cancer survivors but higher than that reported for prostate, colorectal, and melanoma survivors. With regard to mental health, over 40% of our sample reported impairment, which is higher than for these same common cancers. These results are consistent with Schulz et al, who reported that TCS have an HRQoL similar to that of breast cancer patients but poorer than survivors of melanoma or colorectal cancer (32).

When compared with the general French population, physical impairment in our sample was modest, whereas mental health impairment was moderate and clinically meaningful. Nickel et al reported persistently lower HRQoL levels over time among TCS compared with population controls, with substantial impairment in mental health and emotional functioning (33). Collectively, these findings emphasize that TC survivorship entails a considerable long-term HRQoL burden.

Longitudinal analysis revealed that most participants maintained stable PCS and MCS scores over the first 5 years post-diagnosis, indicating minimal clinically meaningful improvement in overall HRQoL. This reflects Husson et al (2012), who reported no significant differences in HRQoL across groups <5, 5-10, and ≥10 years since diagnosis. This result suggests that HRQoL in TCS may reach an early post-diagnosis plateau, with minimal variation thereafter (34).

We also identified several factors associated with long-term HRQoL impairment. Modifiable factors included activity limitation, weight gain, fatigue, anxiety, and treatment-related dissatisfaction with body image, whereas non-modifiable factors were age, sex, and educational level. These findings highlight potential targets for interventions aimed at improving long-term HRQoL in TCS. We describe these findings in detail below.

### Modifiable characteristics

At 5 years post-diagnosis, TCS with activity limitation 5 years post-diagnosis were more likely to have a low PCS score. Forty-six percent of the study sample reported this condition, which reflects recent data showing that up to 52% of TCS remain physically inactive after diagnosis (35). More generally, fewer than 10% of cancer survivors meet physical activity guidelines during treatment (36), spending approximately two-thirds of their time performing sedentary activities (37). Encouraging physical activity is crucial, as it can significantly improve HRQoL by reducing fatigue, supporting function, and enhancing psychological well-being (38).

Weight gain after treatment was also associated with impaired physical health in the present analysis. Previous studies reported postoperative weight gain in TCS (39), attributed to hormonal changes (in particular spontaneous fluctuations in TSH levels that often require repeated levothyroxine dose adjustments), treatment-related fatigue, and metabolic dysregulation (40).

Moreover, concern about one's body image post treatment was significantly associated with impaired mental health in our study. Prior research found that 61.5% of cancer survivors aged between 30 and 39 reported dissatisfaction with their appearance (41). Research on males’ body image, albeit limited, highlights differences between the 2 sexes; women often focus on appearance and weight, whereas men tend to worry more about physical function and strength. However, men can also suffer from muscle dysmorphia or distress due to scarring and disfigurement (42).

At 5 years post-diagnosis, TCS who experienced decreased or absent sexual desire had a lower MCS score. Overall, 32.6% reported reduced sexual desire, which is slightly lower than the 37% found in previous research among DTC patients (43). Research indicates that thyroid disorders may impair sexual function—probably due to the effect of thyroid hormones on sex hormone levels—which in turn influences sexual activity in those affected (44). For example, in women, hypothyroidism is often linked to reduced libido, and low thyroid hormone levels are associated with vaginal dryness, leading to discomfort, especially during intercourse (45). These difficulties also often correlate with depression, body image issues, and reduced intimacy (30). More broadly, sexual dissatisfaction is linked to poorer HRQoL in persons with reproductive or non-reproductive cancers (46). For TCS, delayed resumption of sexual activity may stem from a fear of radioactive contamination, illustrating gaps in communication and psychological support (43).

In our study, 63.6% of patients exhibited anxiety symptoms 5 years after diagnosis, and the presence of suspected or confirmed anxiety was strongly associated with lower PCS scores, highlighting the significant impact of psychological distress on HRQoL. Longitudinal data on persons diagnosed with cancer suggest that psychological vulnerability can be detected at diagnosis, with patients showing early anxiety experiencing poorer baseline HRQoL and further decline over time (47). Collectively, these findings emphasize the importance of early screening and tailored psychosocial interventions to identify and support vulnerable patients from the outset of care.

### Non-modifiable characteristics

Younger survivors reported lower PCS scores, which may reflect factors previously described in the literature concerning this subgroup, including treatment-related effects (eg, RAI and prolonged TSH suppression) and greater vulnerability to psychological and thyroid-related comorbidities (7). However, these factors were not directly assessed in the present study, limiting the understanding of treatment effects. Moreover, men exhibited significantly lower MCS scores than women. This finding aligns with Vitale et al (2024), who reported higher anxiety levels among male cancer survivors (48). Previous research also indicates that men are often diagnosed at a more advanced stage of disease and have higher recurrence rates than women (49). Additionally male TCS have higher mortality rates, are more likely to engage in poor health behaviors (50), and face greater challenges in expressing emotions and seeking help. Hegemonic masculinity may contribute to these barriers, as men are often less likely to pursue psychological support compared to women (51). To our knowledge, only one study to date—included in a systematic review of the benefits of physical activity among TCS—stratified results by sex (11). This highlights the need for sex-specific research to better tailor supportive care interventions.

Lower educational attainment was also significantly associated with lower PCS and MCS in our study, which aligns with previous findings linking low education levels to lower HRQoL in TCS (52). Third level education is generally associated with greater health literacy and more proactive healthcare-seeking behaviors (53). Moreover, in our analysis, survivors with a lower education level were over 3 times more likely to be unemployed, another factor known to negatively affect HRQoL (54). All these findings point to the broader impact of social determinants of health on survivorship.

Finally, although treatment-related variables were not included in the bivariable analyses, existing literature suggests that intensive treatment strategies, such as total thyroidectomy (55), RAI (56), and prolonged TSH suppression, may negatively affect HRQoL (57). Therefore, our findings should be interpreted in the context of evolving clinical practices, with an ever-growing emphasis on treatment de-escalation strategies for low-risk patients.

### Clinical recommendations

Collectively, our study findings highlight the importance of early screening to identify patients who may be more vulnerable to psychosocial difficulties even before TC care begins. Our results suggest that integrating tailored psychosocial support into follow-up pathways could help address these needs more effectively. Beyond the technical surveillance of TC (eg, biological monitoring and imaging), greater involvement of paramedical professionals may be particularly valuable, as ensuring adequate psychosocial support often requires a great deal of time and dedication.

### Strengths and limitations

This study has several strengths. First, we used health insurance data covering private-sector employees and self-employed workers which represent approximately 88% of the French population (58). Accordingly, our findings for TCS can be considered generalizable across France. Second, the use of data collected at 2 key time points—2 and 5 years post-diagnosis—offers valuable insight into how physical and mental HRQoL evolve over time in this population. Third, our analyses were based on a combination of robust data sources, including clinical records and a national medico-administrative database, as well as comprehensive patient-reported outcomes measured with standardized scales. This combination provided us with a comprehensive and multidimensional understanding of survivors’ experiences.

The study also has limitations. First, as with any study using self-reported data, recall and social desirability biases are possible. Second, only participants who could perform a CATI phone interview in French were included; this choice may have excluded subgroups, such as migrants. Third, the lack of data on participants’ ethnicity (information that cannot be collected under French law) constrained our ability to examine possible inter-ethnical differences. Fourth, selection bias cannot be ruled out, as healthier individuals and those who contact healthcare services more frequently may have been more likely to participate, given that they are more engaged with their health and have easier access to medical follow-up. However, no significant differences were observed between participants in VICAN 2 and 5 and decliners in terms of gender or socioeconomic status, which are the main potential sources of selection bias in observational studies. Fifth, while the use of the SF-12 is a major strength of this study, it has limitations. Despite being a widely validated instrument that provides norm-based scores, which enables robust comparisons with the general population, because it is a generic measure assessing symptoms, functional status, and health perceptions, it does not reflect overall HRQoL (59) and may not fully capture the specific concerns of TCS or hidden long-term treatment effects. Moreover, because scores are standardized to population norms, our results reflect relative deviations from the general population rather than absolute levels of HRQoL; this should be considered when interpreting the findings. Sixth, given the moderate sample size, a stepwise selection procedure combining forward and backward approaches was used to identify variables associated with impaired HRQoL. Although TC-specific treatment information was available (total thyroidectomy: 97.6%, RAI: 86.0%, hormone therapy: 96.2%), we excluded it from the final multivariate model because of a lack of treatment variability. We acknowledge that the stepwise selection method may prioritize statistical significance over clinical relevance and may introduce model instability. Therefore, results should be interpreted with caution, and the absence of certain clinically relevant variables in the final models does not necessarily imply a lack of association. Seventh, several factors may act as confounders in the outcome analyses. For instance, although permanent hypoparathyroidism is rare after thyroid surgery, it can cause significant clinical consequences—including chronic hypocalcemia and reduced HRQoL (60)—and should therefore be considered a potential confounding factor missing from this study. Finally, the VICAN 2 and VICAN 5 study cohorts largely reflect treatment practices in the early 2010s, with nearly all patients undergoing total thyroidectomy. Consequently, patients treated with thyroid lobectomy, which is now much more commonly recommended for low-risk disease, are underrepresented. Although this may limit the generalizability of our findings to contemporary patient populations, our work may be beneficial in future research measuring the effects of evolving clinical practices on HRQoL.

## Conclusion

This study demonstrates that 5 years after diagnosis, a considerable proportion of TCS continued to experience physical and, more notably, mental HRQoL impairment. Despite the generally favorable prognosis of TC, these findings underscore that long-term recovery is not synonymous with full restoration of HRQoL. More specifically, the stability of PCS and MCS HRQoL scores between 2 and 5 years post-diagnosis suggests more a plateau effect rather than a gradual recovery. Importantly, modifiable factors such as physical inactivity, weight gain, fatigue, anxiety, and dissatisfaction with one's body image provide actionable targets for supportive care interventions.

More generally, our results emphasize the need to move beyond survival as the sole endpoint in TC care and to integrate long-term, multidisciplinary strategies aimed at improving both physical and mental outcomes. Tailored interventions addressing identified risk factors could play a crucial role in enhancing TC survivors’ well-being and in reducing the persistent burden of the disease.

## Data Availability

The datasets presented in this article are not readily available because of the sensitive nature of the data and possible high risks associated with patient confidentiality. Requests to access the datasets should be directed to anne-deborah.bouhnik@inserm.fr.
